# Use of Gas Chromatography-Mass Spectrometry Techniques (GC-MS, GC-MS/MS and GC-QTOF) for the Characterization of Photooxidation and Autoxidation Products of Lipids of Autotrophic Organisms in Environmental Samples

**DOI:** 10.3390/molecules27051629

**Published:** 2022-03-01

**Authors:** Jean-François Rontani

**Affiliations:** Mediterranean Institute of Oceanography (MIO), Aix Marseille University, Université de Toulon, CNRS, IRD, UM 110, 13288 Marseille, France; jean-francois.rontani@mio.osupytheas.fr; Tel.: +33-(0)4-86-09-06-02

**Keywords:** senescent phototrophs, unsaturated lipids, photooxidation, autoxidation, gas chromatography-mass spectrometry, specific tracers, TMS derivatives, EI fragmentation, environment

## Abstract

This paper reviews applications of gas chromatography-mass spectrometry techniques for the characterization of photooxidation and autoxidation products of lipids of senescent phototrophic organisms. Particular attention is given to: (i) the selection of oxidation products that are sufficiently stable under environmental conditions and specific to each lipid class and degradation route; (ii) the description of electron ionization mass fragmentation of trimethylsilyl derivatives of these compounds; and (iii) the use of specific fragment ions for monitoring the oxidation of the main unsaturated lipid components of phototrophs. The techniques best geared for this task were gas chromatography-quadrupole-time of flight to monitor fragment ions with very high resolution and accuracy, and gas chromatography-tandem mass spectrometry to monitor very selective transitions in multiple reaction monitoring mode. The extent of the degradation processes can only be estimated if the oxidation products are unaffected by fast secondary oxidation reactions, as it is notably the case of ∆^5^-sterols, monounsaturated fatty acids, chlorophyll phytyl side-chain, and di- and triterpenoids. In contrast, the primary degradation products of highly branched isoprenoid alkenes possessing more than one trisubstituted double bond, alkenones, carotenoids and polyunsaturated fatty acids, appear to be too unstable with respect to secondary oxidation or other reactions to serve for quantification in environmental samples.

## 1. Introduction

Phototrophic organisms (mainly green plants, algae, cyanobacteria and some protists) carry out photosynthesis that is, conversion of sunlight energy, carbon dioxide and water into organic materials. Due to the generation of highly reactive oxygen species (ROS) during photosynthetic electron transport, these organisms are particularly sensitive to oxidative damages [1]. Lipids (hydrocarbons, pigments, terpenoids, free fatty acids, acylglycerides, phospholipids, galactolipids, cutins, suberins and waxes [2]) are important components of phototrophic organisms, accounting for 16–26% of organic content in phytoplankton [3] and up to 45% in the green alga *Botryococcus Braunii* [4]. The relative stability and specificity of lipids makes them popular tracers of the origin of organic matter in environmental samples [5,6,7]. Their abiotic oxidation products can be also very useful for estimating present or past photooxidative and autoxidative alterations in specific phototrophic organisms [8,9].

The most common chromatographic methods for lipid analysis are gas chromatography (GC), and high-performance liquid chromatography (HPLC) coupled with mass spectrometers (MS). GC-based analytical procedures require analytes that are volatile and thermally stable. In practice, this means that GC-based analysis of the oxidation products of mixtures of complex and simple lipids with such techniques demands a chemical pre-treatment of the samples, including: (i) NaBH_4_ reduction of thermally-labile hydroperoxides to the corresponding alcohols [10], (ii) alkaline hydrolysis of complex lipids into their constituent fatty acids, plus glycerol, phosphate, sterol or sugar groups [5], and then (iii) conversion of polar compounds to volatile derivatives (derivatization). Despite this added time-consuming pre-treatment (which is not necessary with HPLC-MS analyses), GC-MS techniques involving electron ionization (EI) and chemical ionization (CI) are widely employed for the characterization of lipid oxidation products [11,12,13]. Indeed, EI provides more structural information than the soft ionization techniques such as electron spray ionization (ESI) or atmospheric pressure chemical ionization (APCI) employed in HPLC-MS analyses, notably as it enables easy determination of the position of functional groups of lipid oxidation products [14]. However, the relatively soft ESI and APCI ionization modes used during HPLC-MS analyses allow structural characterization of thermally-labile compounds (e.g., hydroperoxides) [15]. Moreover, the possibility to work in reverse-phase liquid chromatography also allows the analysis of compounds too heavy to be amenable by GC (e.g., triacylglycerides) [15,16]. Note that other powerful non-chromatographic techniques such as matrix-assisted laser desorption/ionization mass spectrometry (MALDI-MS) [17,18], ion-mobility mass spectrometry (IM-MS) [19,20], and nuclear magnetic resonance (NMR) [21] also appeared to be very useful for the characterization of lipid oxidation products. 

In this review, particular attention is given to the use of gas chromatography-tandem mass spectrometry (GC-MS/MS) and gas chromatography-quadrupole-time of flight (GC-QTOF) techniques for the characterization of trimethylsilyl (TMS) derivatives of lipid oxidation products in senescent phototrophic organisms. GC-MS/MS can perform analyses in multiple reaction monitoring (MRM) mode based on specific collision-induced fragmentations of precursor ions, which substantially increase signal-to-noise ratios and method sensitivity [22]. GC-QTOF offers high mass resolution and accuracy and can use narrow mass intervals reducing interferences and background noise, making it particularly suitable for identifying unknown lipid oxidation products in complex natural extracts.

Trimethylsilylation is the method most commonly employed for derivatization of lipids in GC-MS analyses [23,24]. TMS derivatives are produced by replacing the active hydrogen atom of alcohols, acids, amines and thiols by a trimethylsilyl group. These derivatives are highly volatile, thermally stable and present outstanding gas chromatographic characteristics. EI mass spectra of TMS derivatives generally exhibit a significant [M − 15]^+^ ion formed by loss of a silicon-bonded methyl group, which is especially useful for determining molecular mass. Fragmentations of these derivatives are also hugely informative for structural elucidations [25,26].

## 2. Abiotic Oxidation of Lipid Components of Autotrophic Organisms

### 2.1. Type II Photosensitized Oxidation

Due to the presence of chlorophyll, which is a very efficient photosensitizer [27,28], visible light-induced photosensitized processes act intensively during the senescence of autotrophic organisms. In healthy cells, the excited singlet state of chlorophyll (^1^Chl) formed after absorption of a quantum of light energy, leads predominantly to the characteristic fast photosynthesis reactions [27]. However, a small proportion of ^1^Chl undergoes intersystem crossing (ISC) to form the longer live triplet state (^3^Chl) [28], which is not only itself potentially damaging in type I reactions [28] but can also generate ROS and, in particular, singlet oxygen (^1^O_2_) by reacting with ground state oxygen (^3^O_2_) (type II processes). As a defense against oxidative damage, there are many antioxidant compounds (e.g., carotenoids and vitamin E) and enzymes (e.g., superoxide dismutase and catalase) that operate in chloroplasts [27,29].

As fast photosynthesis reactions are clearly not operative in senescent phototrophic organisms, potentially damaging ^3^Chl and ^1^O_2_ [30] are produced at an accelerated rate exceeding the quenching capacity of the photoprotective system and thus damage the membranes (photodynamic effect [31]). ^1^O_2_ readily oxidizes cellular components of senescent autotrophic organisms such as unsaturated lipids (including ∆^5^-sterols, unsaturated fatty acids, chlorophyll phytyl side-chain, carotenoids and alkenes), proteins, and nucleic acids [32]. The rate of reaction of ^1^O_2_ with olefins is controlled by the degree of substitution and the configuration (*cis*- or *trans*-) of the double bond [33], with highly-substituted and *cis*- double bonds being the more reactive. Type II photosensitized oxidation of unsaturated lipids affords allylic hydroperoxides (for reviews see [8,9]).

### 2.2. Free Radical Oxidation (Autoxidation)

Due to spin restriction [34], the unpaired electrons of ground-state triplet molecular oxygen ^3^O_2_ can only interact with unpaired electrons of organic radicals, which drive autoxidation reactions. Autoxidation involves free-radical-mediated oxidation chain reactions, which can be divided into three steps: chain initiation, propagation, and termination [35]. Initiation of autoxidation requires initiators that are able to produce radicals by removing an electron to the substrate molecule or breaking a covalent bond. The most common initiators are heat, light, redox-active metal ions undergoing one-electron transfer (e.g., Fe^2+^, Co^2+^, Fe^3+^, Cu^2+^, Mn^2+^, Zn^2+^, Mg^2+^, V^2+^), and certain enzymes (lipoxygenases). The propagation step involves a succession of reactions in which each radical produced in one reaction is consumed in the next [36]. It generally proceeds via: (i) hydrogen atom abstraction from tertiary, allylic or α to oxygen positions, and (ii) addition of peroxyl radicals to double bonds. Termination results from reactions of radicals affording non-radical products. In senescent phototrophic cells, initiation of autoxidation processes is generally attributed to the cleavage (induced by heat, light, metals or enzymes) of hydroperoxides resulting from type II photosensitized oxidation of cellular components to hydroxyl, peroxyl and alkoxyl radicals [37,38].

## 3. Characterization of the Oxidation Products of Lipids

This chapter briefly describes the mechanisms of photooxidation and autoxidation of the main unsaturated lipids of phototrophic organisms. A focus is given to the selection of oxidation products sufficiently stable and specific to act as tracers of these processes in environmental samples (such as: phytodetritus, particulate matter, marine and lacustrine sediments and soil), as well as to the mechanisms of fragmentation of TMS derivatives of these compounds during electron ionization. Some application examples of these tracers are also shown. Note that accurate masses of the different fragment ions formed are given, which makes them amenable to use in GC-QTOF analyses, while the corresponding unit masses can still be used in GC-MS/MS or classical GC-MS analyses.

### 3.1. Chlorophyll Phytyl Side-Chain

**Scheme 1:**
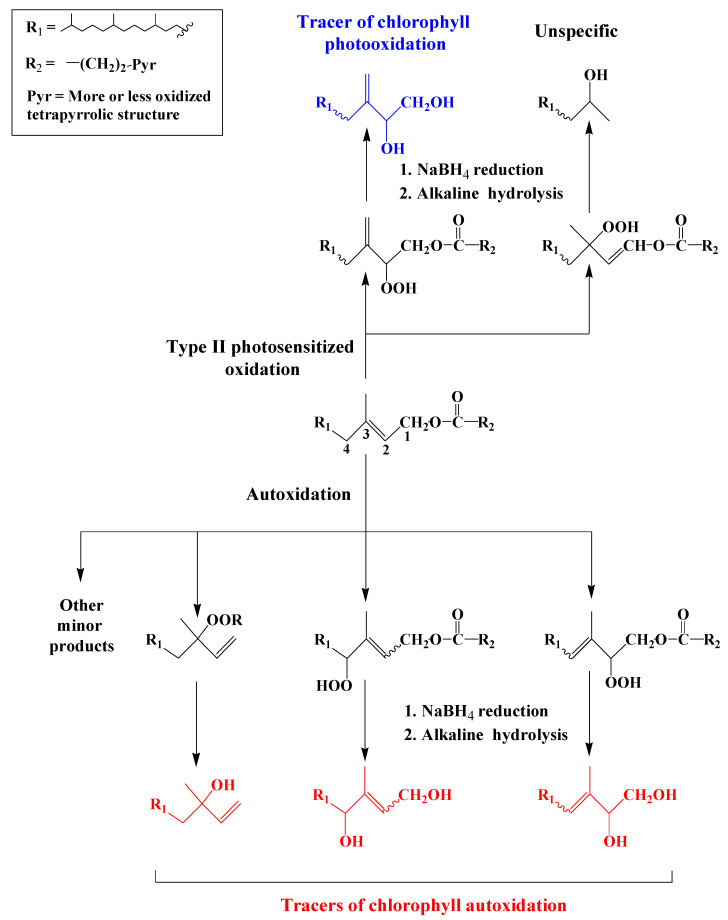
Photooxidation and autoxidation of chlorophyll phytyl side-chain.

Attack of ^1^O_2_ on the tri-substituted double bond of the chlorophyll phytyl side-chain affords two allylic hydroperoxides, which may be recovered in the form of 6,10,14-trimethylpentadecan-2-ol and 3-methylidene-7,11,15-trimethylhexadecan-1,2-diol (phytyldiol) after NaBH_4_ reduction and alkaline hydrolysis [39] (Scheme 1). The stable and highly specific phytyldiol was proposed as biogeochemical marker of chlorophyll photodegradation in the natural environment [40]. In contrast, free radical oxidation (autoxidation) of chlorophyll phytyl side-chain and subsequent reduction and hydrolysis gives 3,7,11,15-tetramethylhexadec-3-en(*Z*/*E*)-1,2-diols, 3,7,11,15-tetramethyl-hexadec-2-en(*Z*/*E*)-1,4-diols and 3,7,11,15-tetramethyl-hexadec-1-en-3-ol (isophytol) [41,42] (Scheme 1). These compounds have been proposed as specific tracers of chlorophyll phytyl side-chain autoxidation in environmental samples [41,42].

**Scheme 2:**
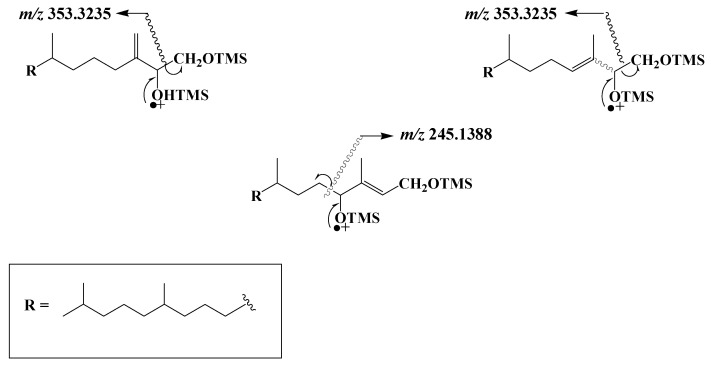
Main EI mass fragmentations of TMS derivatives of phytyldiol, 3,7,11,15-tetramethylhexadec-3-en(*Z*/*E*)-1,2-diols and 3,7,11,15-tetramethyl-hexadec-2-en(*Z*/*E*)-1,4-diols.

TOF mass spectra of the TMS derivatives of phytyldiol and 3,7,11,15-tetramethylhexadec-3-en(*Z*/*E*)-1,2-diols show intense and specific fragment ions at *m*/*z* 353.3235 resulting from classical α-cleavage between the carbon atoms 1 and 2 bearing the two TMS ether groups [31], while the spectra of TMS derivatives of 3,7,11,15-tetramethyl-hexadec-2-en(Z/E)-1,4-diols are dominated by a fragment ion at *m*/*z* 245.1388 corresponding to α-cleavage between carbon atoms 4 and 5 (Scheme 2).

**Figure 1:**
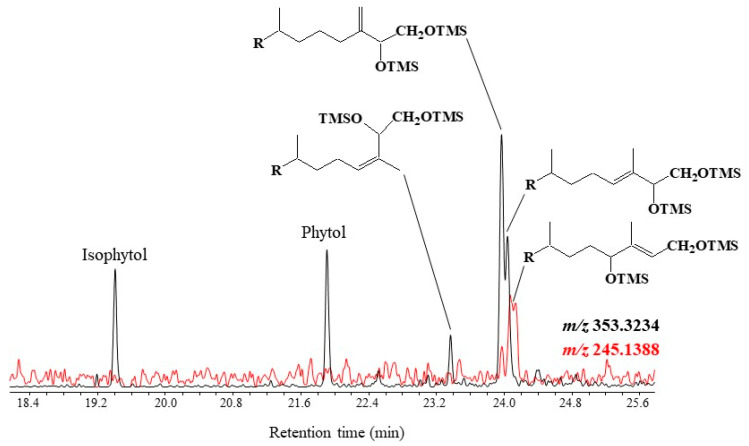
Partial TOF ion chromatograms (*m*/*z* 353.3235 and 245.1388) showing the presence of TMS derivatives of phytol and its main photooxidation and autoxidation products in senescent cells of the diatom *Thalassiosira* sp.

Note that the loss of a methyl radical by the molecular ion of TMS derivatives of phytol and isophytol also affords a fragment ion at *m*/*z* 353.3235. Monitoring ions at *m*/*z* 353.3235 and 245.1388 thus allows simultaneous characterization and quantification of phytol and its main photooxidation and autoxidation products in natural samples (see example given in Figure 1). 

### 3.2. ∆^5^-Sterols

**Scheme 3:**
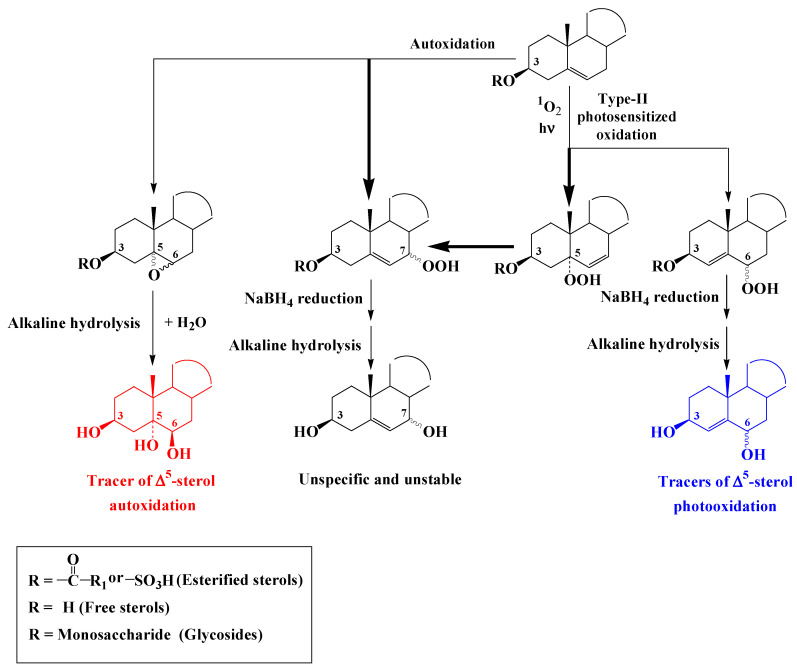
Photooxidation and autoxidation of ∆^5^-sterols.

Reaction of ^1^O_2_ with the double bond of ∆^5^-sterols mainly affords a ∆^6^-5α-hydroperoxide and to a lesser extent ∆^4^-6α/β-hydroperoxides [43,44] (Scheme 3). Under environmental conditions ∆^6^-5α-hydroperoxide undergoes fast allylic rearrangement to unstable and unspecific 7α/β-hydroperoxides (Scheme 3). ∆^4^-Stera-3β,6α/β-diols resulting from NaBH_4_-reduction and alkaline hydrolysis of ∆^4^-6α/β-hydroperoxides were thus proposed as specific tracers of type II photosensitized oxidation of the corresponding ∆^5^-sterols [45,46].

Autoxidation of ∆^5^-sterols mainly affords unstable and unspecific 7α/β-hydroperoxides after hydrogen atom abstraction at the allylic carbon atom 7 [47]) (Scheme 3). Smaller proportions of isomeric 5α,6α- and 5β,6β-epoxysterols are also produced after addition of peroxyl radical to the double bond [48] (Scheme 3). Stable and specific 3β,5α,6β-trihydroxysterols resulting from the hydrolysis of these epoxides during alkaline hydrolysis and in environmental conditions were proposed as specific tracers of the autoxidation of ∆^5^-sterols [45,46].

**Scheme 4:**
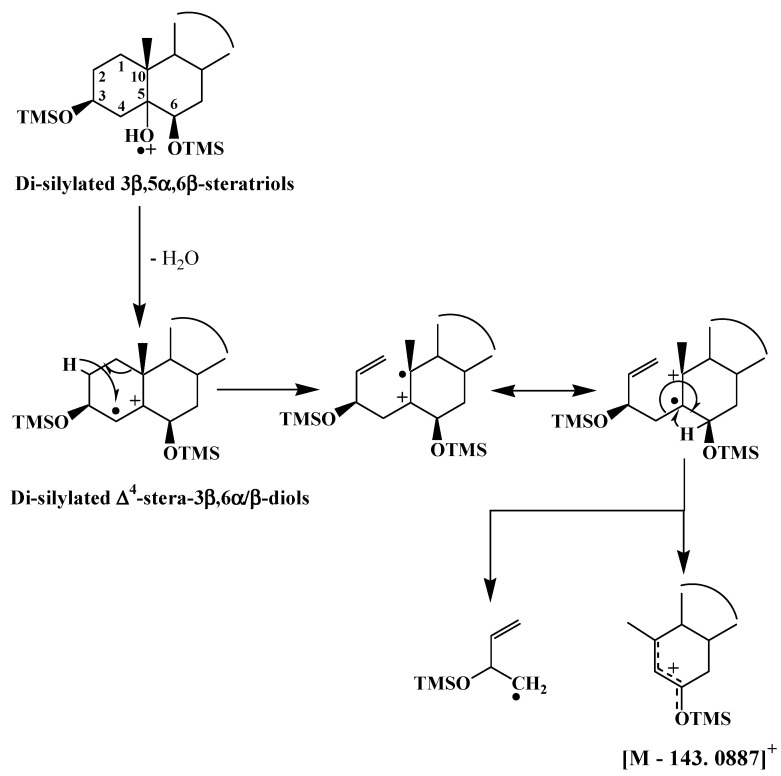
Proposed formation pathways of the fragment ion [M − 143.0887]^+^ in TOF mass spectra of ∆^4^-stera-3β,6β-diol and 3β,5α,6β-trihydroxysterol TMS derivatives.

TOF mass spectra of ∆^4^-stera-3β,6α/β-diol TMS derivative exhibit an intense and interesting fragment ions at [M − 143.0887]^+^ resulting from double bond ionization and subsequent hydrogen migrations and cleavages of the C_1_–C_10_ and C_4_–C_5_ bonds [49] (Scheme 4). Due to steric hindrance, the classical silylation reagents only silylate 3β,5α,6β-trihydroxysterols to their 3 and 6 positions [50], and during ionization their TMS derivatives very easily lose a neutral molecule of water and thus exhibit mass spectra that are very similar to those of ∆^4^-stera-3β,6β-diol TMS derivatives (Scheme 4).

**Figure 2:**
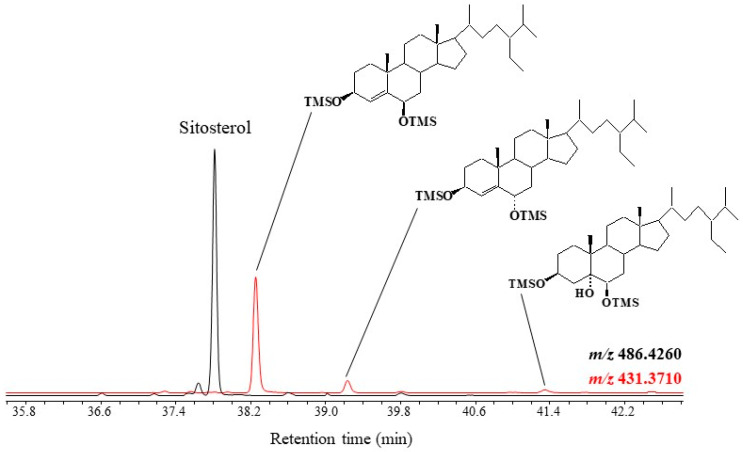
Partial TOF ion chromatogram (*m*/*z* 431.3710 and 486.4260) showing the presence of TMS derivatives of 24-ethylcholest-5-en-3β-ol (sitosterol) ([M]^+•^ = 486.4260) and its photo- ([M − 143.0887]^+^ = 431.3710) and autoxidation ([M − H_2_O − 143.0887]^+^ = 431.3710) products in senescent leaves of *Smilax aspera*.

Specific fragment ions [M − 143.0887]^+^, for which Table 1 gives accurate masses for the ∆^4^-stera-3β,6β-diols of the more common sterols, thus emerged as very useful for the monitoring of ∆^5^-sterol photooxidation and autoxidation in phototrophic organisms. An example of their application is given in Figure 2.

### 3.3. Unsaturated Fatty Acids

Type II photosensitized oxidation and autoxidation rates of unsaturated fatty acids logically increase with their number of double bonds [51,52]. Unfortunately, oxidation products of the more reactive polyunsaturated fatty acids (PUFAs) are not sufficiently stable under environmental conditions to be used as tracers of these degradation processes in situ. Note that isoprostanoids (cyclopentane-containing oxylipins) resulting from autoxidation of C_18_, C_20_ and C_22_ PUFAs are often used as biomarkers for in vivo oxidative stress in animals and plants [53]. These compounds could be detected in higher plants and algae by using GC–MS in negative-ion chemical ionization (NICI) mode [53,54]. However, in the literature there are no reports of such compounds in environmental samples. 

**Scheme 5:**
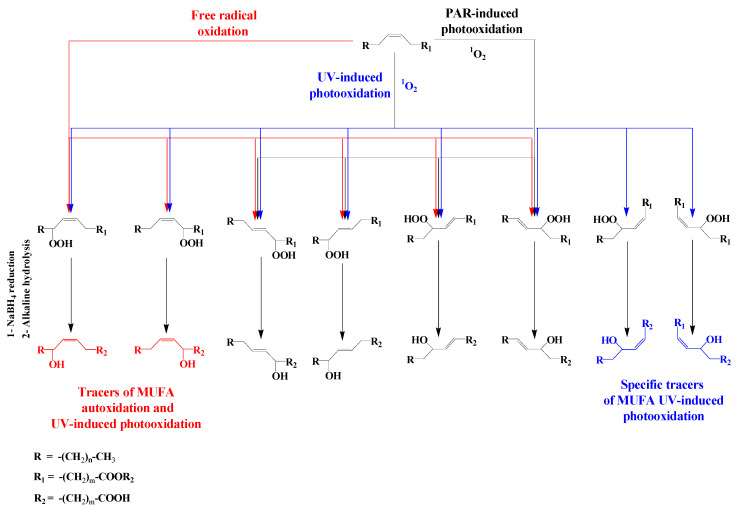
Type II photosensitized oxidation (induced by PAR and UV radiations) and autoxidation of MUFAs.

In contrast, oxidation products of monounsaturated fatty acids (MUFAs) are sufficiently stable for use as tracers of type-II photosensitized oxidation and autoxidation processes in situ [10]. During type II photosensitized oxidation of MUFAs, attack by ^1^O_2_ of the two ethylenic carbon atoms of the double bond leads to the formation of two *trans*- allylic hydroperoxides [52,55], which subsequently undergo stereoselective radical allylic rearrangement to afford two other isomers with a *trans*-double bond [56] (Scheme 5). Note that if type II photosensitized oxidation of MUFAs involves UV radiation, then four corresponding *cis*-allylic hydroperoxides also get produced [57] (Scheme 5). In contrast, free radical oxidation of these compounds affords only two *cis*-allylic hydroperoxides (corresponding to the oxidation of the two allylic positions of MUFAs) in addition to the four *trans*-isomeric hydroperoxides [56] (Scheme 5). Consequently, in senescent autotrophic organisms a dominance of these two *cis*-isomers (among the four *cis*-isomers) points to the involvement of autoxidation processes, while a dominance of all four *cis*-isomers points to UV-induced photodegradation.

**Scheme 6:**
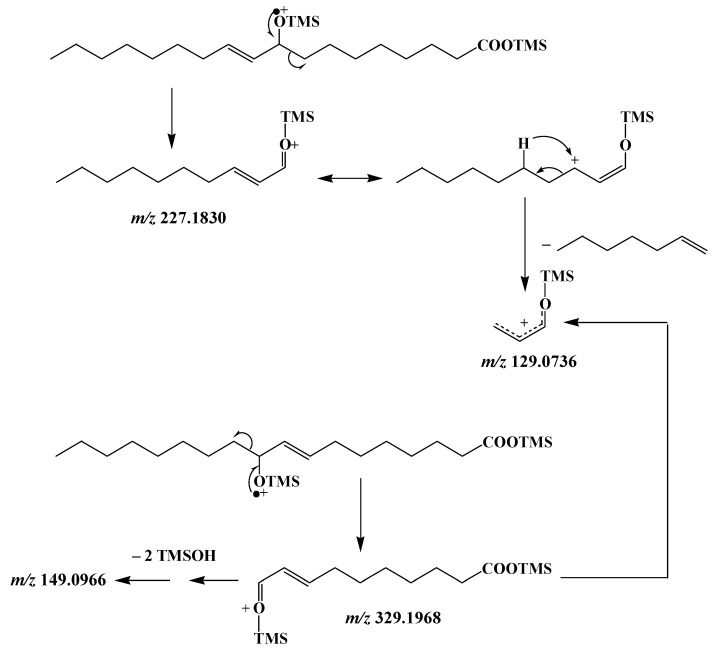
Examples of EI fragmentations of TMS derivatives of MUFA oxidation products.

Under EI, TMS derivatives of isomeric allylic hydroxy acids resulting from photooxidation and autoxidation of MUFAs and subsequent NaBH_4_ reduction undergo α-cleavage at their TMS ether group. Cleavage acts on the saturated side of the molecule (as the vinylic position of the double bond hinders cleavage on the other side) and affords stable and specific fragment ions (Scheme 6) that are dependent on the carbon atom number and double-bond position of the MUFA considered [11,15]. The fragment ions resulting from α-cleavage of silylated oxidation products of the more common MUFAs are listed in Table 2.

**Figure 3:**
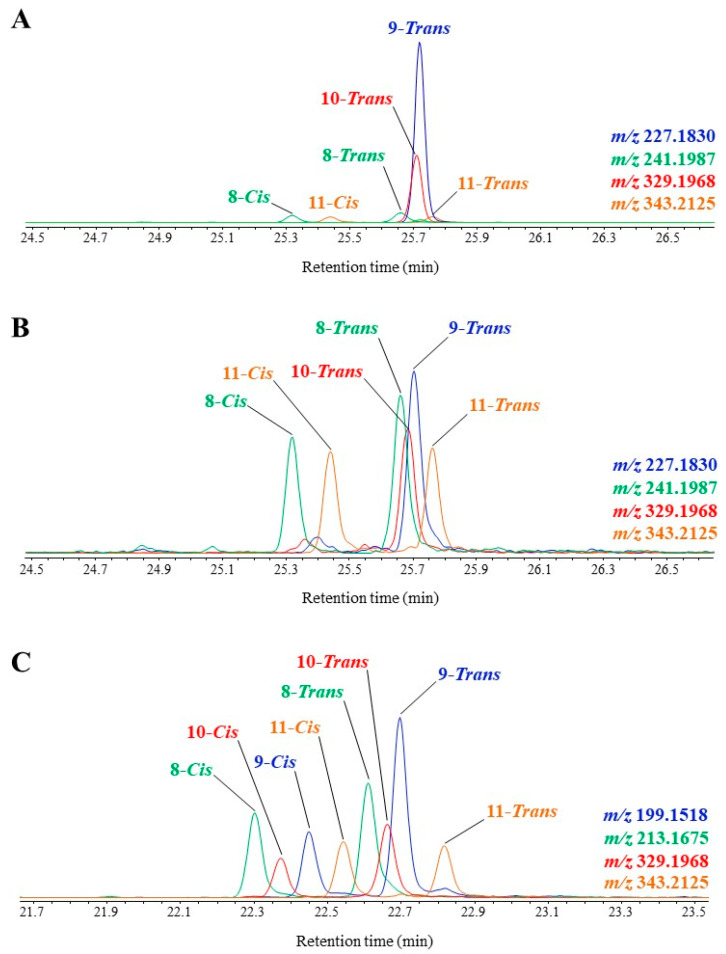
Partial TOF ion chromatograms showing TMS derivatives of MUFA oxidation products in senescent cells of the haptophyte *Emiliania huxleyi* irradiated by visible light (**A**), and after aging (**B**), and of the diatom *Thalassiosira* sp. irradiated by (visible + UV) light (**C**).

GC-QTOF allows a clean characterization and quantification of TMS derivatives of MUFA oxidation products in autotrophic organisms and environmental samples. Figure 3 gives some examples of the technique application showing typical profiles of visible light-induced, (visible + UV) light-induced and autoxidative degradation products.

**Figure 4:**
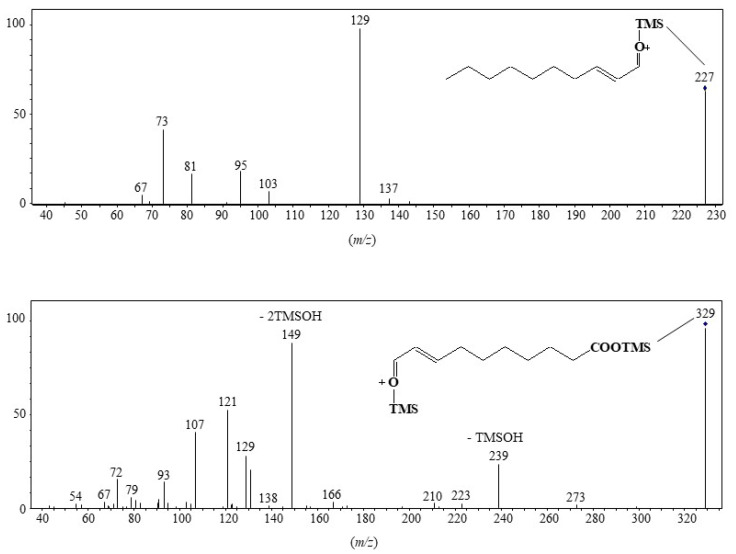
Low energy collision-induced dissociation (CID)-MS/MS (5 eV) of fragment ions at *m*/*z* 227 and 329.

**Figure 5:**
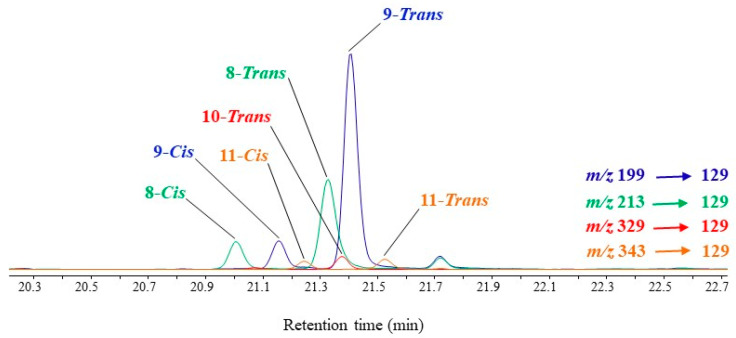
MRM chromatogram (*m*/*z* 199 → 129, *m*/*z* 213 → 129, *m*/*z* 329 → 149 and *m*/*z* 343 → 163) showing the presence of TMS derivatives of palmitoleic acid (C_16:1ω9_) oxidation products in senescent cells of *Thalassiosira* sp. irradiated by sunlight.

MRM analyses of TMS derivatives of MUFA oxidation products involve intense and selective transitions from the ions resulting from α-cleavage (precursor ions) to the fragment ion at *m*/*z* 129 (product ion) (Scheme 6). Note that this transition is more efficient with precursor ions containing the terminal methyl group than with precursor ions containing the TMS ester group, which can easily lose neutral TMSOH molecules (Scheme 6, Figure 4). Figure 5 gives an example of how MRM analyses can be applied.

### 3.4. Pentacyclic Triterpenes

Pentacyclic triterpenes and their derivatives, which are widely found in angiosperms [58], are divided into three main classes, that is, lupanes, oleananes and ursanes.

#### 3.4.1. Lupanes

**Scheme 7:**
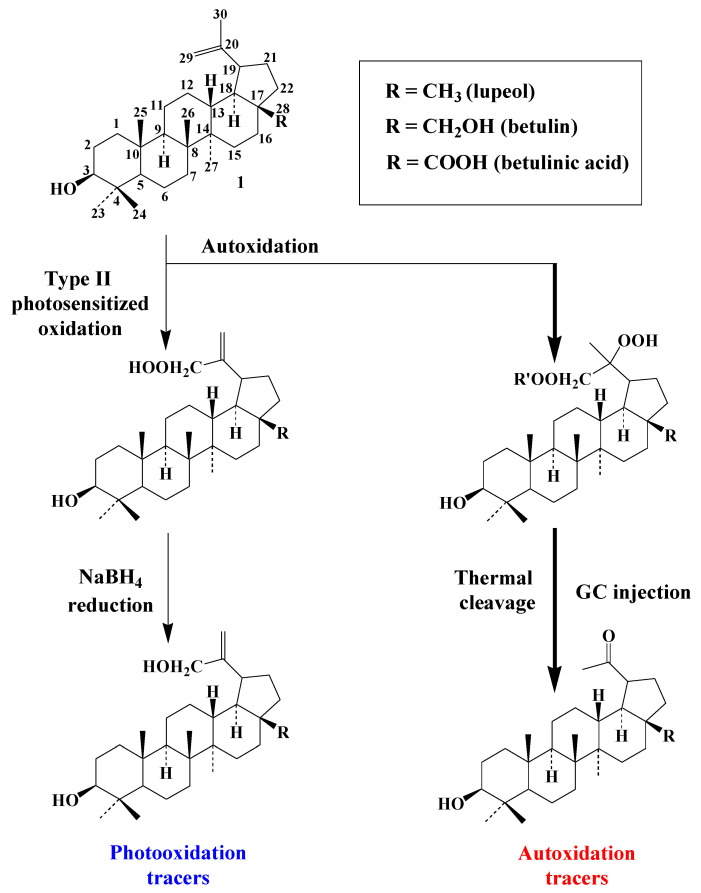
Photooxidation and autoxidation of lupanes.

Type-II photooxidation and autoxidation of lupanes have so far only been studied for betulin [59], but the results obtained can be extended to lupeol or betulinic acid (the main triterpenoids with betulin of the lupane group). ^1^O_2_ reacts slowly with the C_20_–C_29_ double bond of betulin and specifically produces lup-20(30)-ene-3β,28-diol-29-hydroperoxide, which can be quantified after NaBH_4_ reduction in the form of lup-20(30)-ene-3β,28,29-triol (Scheme 7). Lup-20(30)-ene-3β,28,29-triol, lup-20(30)-ene-3β,29-diol (arising from lupeol) and lup-20(30)-ene-3β,29-diol-28-oic acid (arising from betulinic acid) constitute useful specific tracers of photooxidation of lupanes in angiosperms.

In contrast, the autoxidation of betulin mainly involves peroxyl radical addition to the C_20_–C_29_ double bond and mainly affords a diperoxide that is unaffected by NaBH_4_ reduction and converted to stable lupan-20-one-3β,28-diol during hot GC injection [59] (Scheme 7). Lupan-20-one-3β,28-diol, lupan-20-one-3β-ol (arising from lupeol) and lupan-20-one-3β-ol-28-oic acid (arising from betulinic acid) can be used as specific tracers of the autoxidation of lupanes in angiosperms [59,60].

**Scheme 8:**
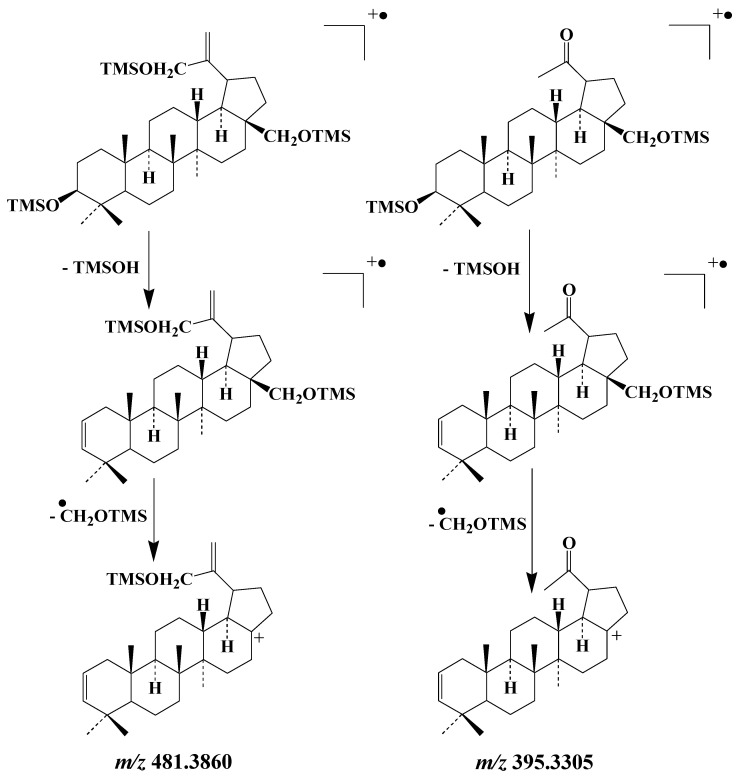
Main EI fragmentations of the TMS derivatives of lup-20(30)-ene-3β,28,29-triol and lupan-20-one-3β,28-diol.

**Figure 6:**
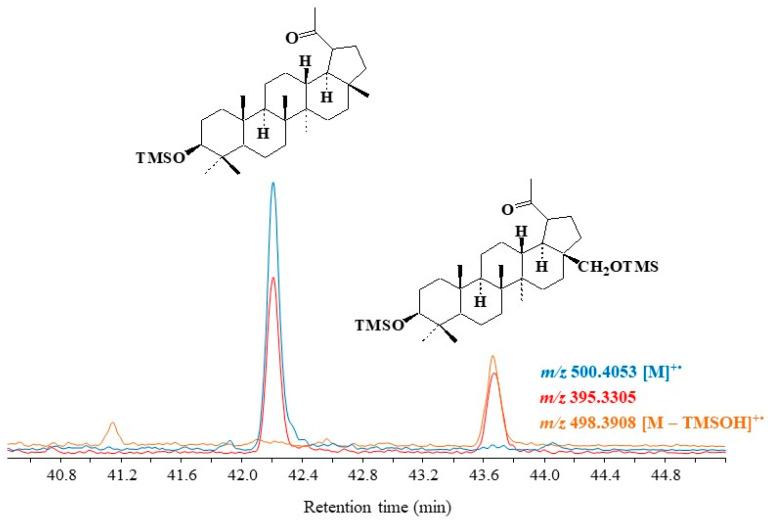
Partial TOF ion chromatograms (*m*/*z* 395.3305, 498.3908 and 500.4053) showing the presence of autoxidation products of lupeol and betulin in higher plant debris collected in the Rhône River.

The EI mass spectra of the TMS derivatives of lup-20(30)-ene-3β,28,29-triol and lupan-20-one-3β,28-diol exhibit intense fragment ions at *m*/*z* 481.3860 and *m*/*z* 395.3305, respectively, whose formation involves elimination of a neutral molecule of TMSOH and subsequent loss of the CH_2_OTMS group borne by the carbon 28 [59] (Scheme 8). These fragment ions make good candidates for monitoring type-II photosensitized oxidation and autoxidation of betulin, respectively, in environmental samples. As the formation of these ions involves the loss of the group borne by carbon 28, they can be also used as tracers of the oxidation of lupeol and betulinic acid. Figure 6 gives an example of the specific fragment ion at *m*/*z* 395.3305 applied for monitoring lupane autoxidation in environmental samples.

#### 3.4.2. Ursanes and Oleanes

**Scheme 9:**
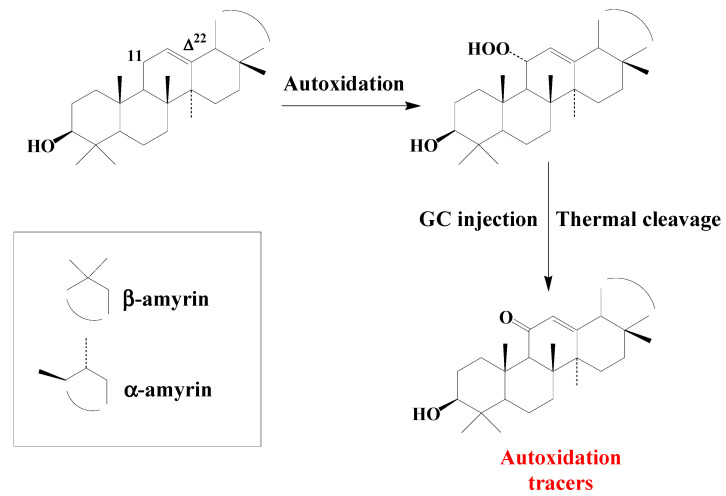
Autoxidation of α- and β-amyrins.

Studies on type II photosensitized oxidation and autoxidation of ursanes and oleanes have mainly focused on α- and β-amyrins [61]. α- and β-amyrins were found to be totally unaffected during photodegradation experiments, due to steric hindrance preventing ^1^O_2_ reaction with their double bond [61]. Autoxidation of amyrins mainly involves hydrogen abstraction and specifically produces 11α-hydroperoxyamyrins [61] (Scheme 9). 

These hydroperoxides, which appeared to be unaffected by NaBH_4_ reduction, are thermally cleaved to the corresponding 11-oxoamyrins during GC or GC-MS analyses using hot injectors (Scheme 9). 11-Oxoamyrins are sufficiently stable and specific to serve as tracers of amyrin autoxidation in senescent angiosperms or environmental samples.

**Scheme 10:**
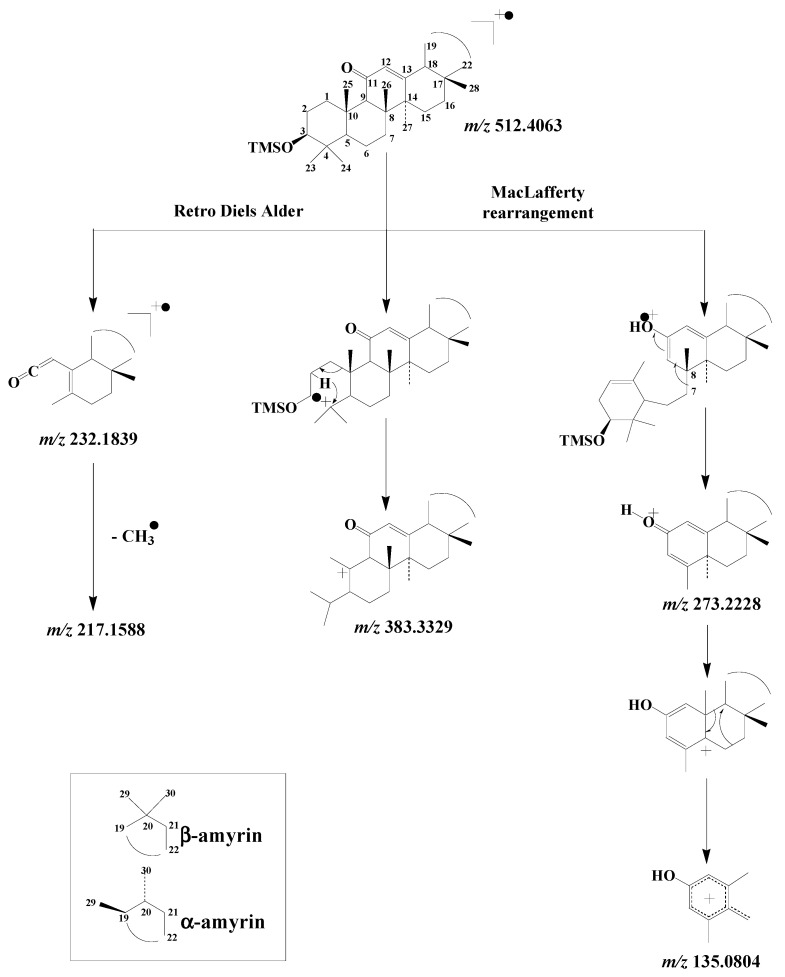
Main EI mass fragmentation of TMS derivatives of 11-oxo-amyrins.

EI fragmentation of TMS derivatives of 11-oxoamyrins was recently studied [62] and found to involve: (i) retro-Diels-Alder cleavage of the unsaturated ring C leading to the formation of a fragment ion at *m*/*z* 232.1822, (ii) γ-hydrogen rearrangement of the ionized 11-keto group and subsequent cleavage of the 7–8 bond affording a well-stabilized fragment ion at *m*/*z* 273.2213, and (iii) a fragmentation pathway involving loss of the TMS group together with carbon atoms 1, 2 and 3 of the A ring after initial cleavage of the 3–4 bond [25] producing a fragment ion at *m*/*z* 383.3308 (Scheme 10). Subsequent fragmentation of the ion at *m*/*z* 273.2213 affords a strongly stabilized ion at *m*/*z* 135.0804 after migration of the methyl group 27 from carbon 14 to carbon 13 and concerted cleavage of the 13–18 and 15–16 bonds [63] (Scheme 10). 

Note that after the loss of a methyl radical, a fragment ion at *m*/*z* 217.1588 can be formed from the fragment ion at *m*/*z* 232.1822 (Scheme 10). This fragmentation, which is more intense in the case of 11-oxo-β-amyrin due to the thermodynamically-favored loss of a methyl radical from the tertiary carbon 20, may be useful for differentiating 11-oxo-amyrins.

**Figure 7:**
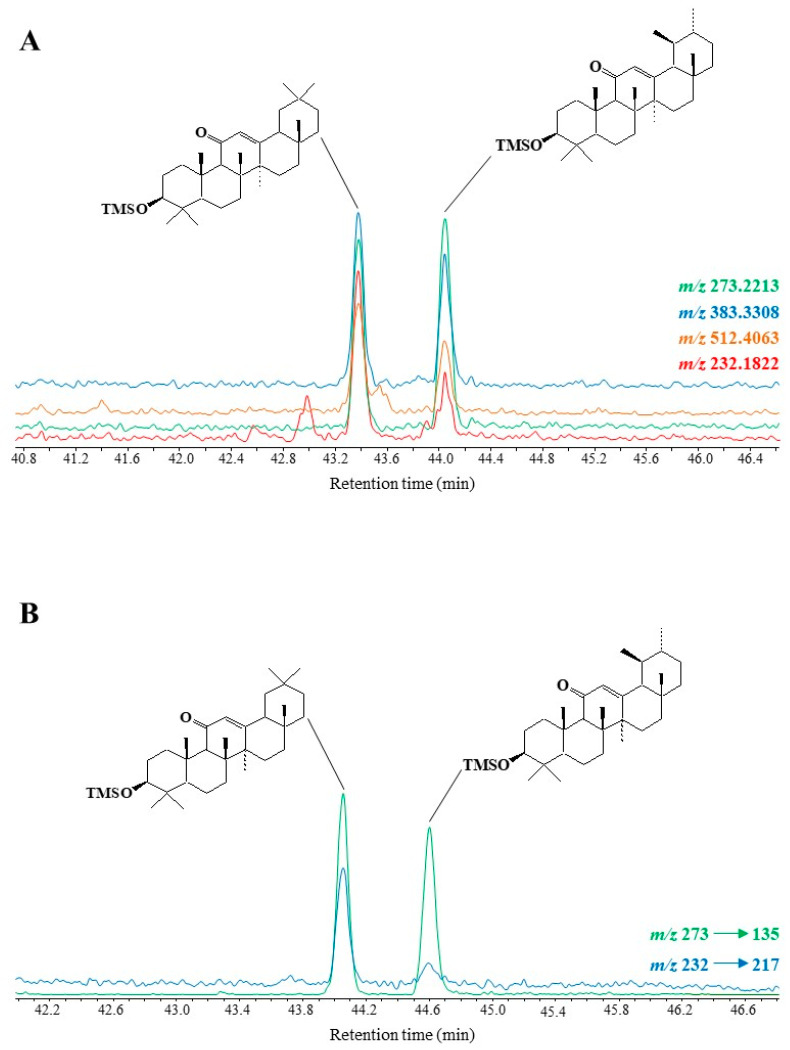
Partial TOF ion chromatogram (*m*/*z* 512.4063, 383.3308, 273.2213 and 232.1822) (**A**) and MRM chromatogram (*m*/*z* 273 → 135 and *m*/*z* 232 → 217) (**B**) showing the presence of oxidation products of amyrins in senescent leaves of *Quercus ilex*.

Specific fragment ions at *m*/*z* 512.4063 [M]^+•^, 383.3308, 273.2213 and 232.1822 appeared to be useful for GC-QTOF monitoring of TMS derivatives of 11-oxo-amyrins in environmental samples (see example given in Figure 7A). MRM analyses using the transitions *m*/*z* 273 → 135 and *m*/*z* 232 → 217 also appeared to be well suited to the detection of traces of these compounds (see Figure 7B).

### 3.5. Dehydroabietic Acid

**Scheme 11:**
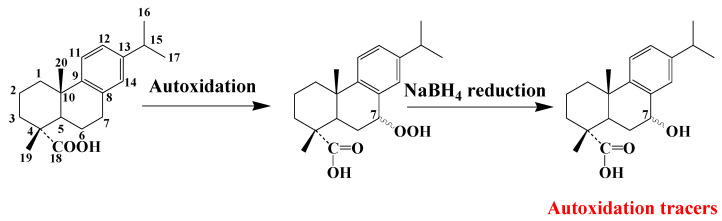
Autoxidation of dehydroabietic acid.

Dehydroabietic acid (8,11,13-abietatrien-18-oic acid), a component of conifers, has long be used as a tracer of gymnosperms [64,65]. Autoxidation of this compound involves hydrogen atom abstraction at the benzylic carbon atom 7 to give 7α/β-hydroperoxydehydroabietic acids [66], which are reduced to the corresponding hydroxy acids during NaBH_4_-reduction (Scheme 11). 7α/β-hydroxydehydroabietic acids are useful tracers of dehydroabietic autoxidation in gymnosperms.

**Scheme 12:**
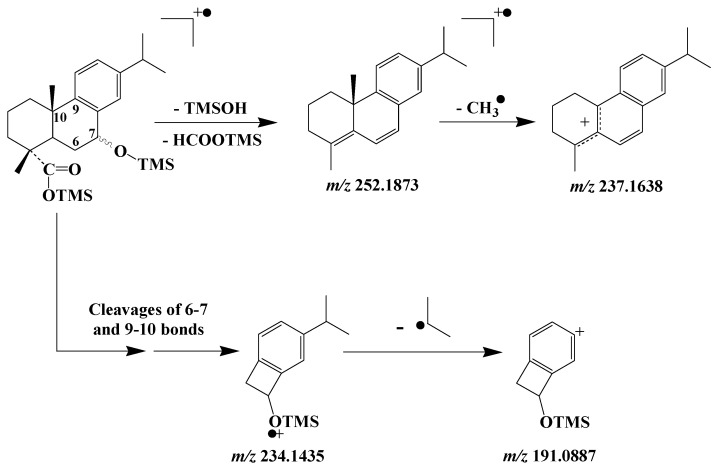
Main EI mass fragmentations of TMS derivatives of 7α/β-hydroxydehydroabietic acids.

EI mass spectra of TMS derivatives of 7α/β-hydroxydehydroabietic acids exhibit intense fragment ions at *m*/*z* 191.0887, 234.1435 and 237.1638 [66]. The formation of the ion at *m*/*z* 237.1638 results from successive losses of neutral TMSOH and formate molecules and subsequent loss of a methyl radical (Scheme 12). The bicyclic fragment ion at *m*/*z* 234.1435 results from complex fragmentation processes involving cleavage of the 6–7 and 9–10 bonds [66] (Scheme 12); and it can readily lose an isopropyl radical to give a stable fragment ion at *m*/*z* 191.0887.

**Figure 8:**
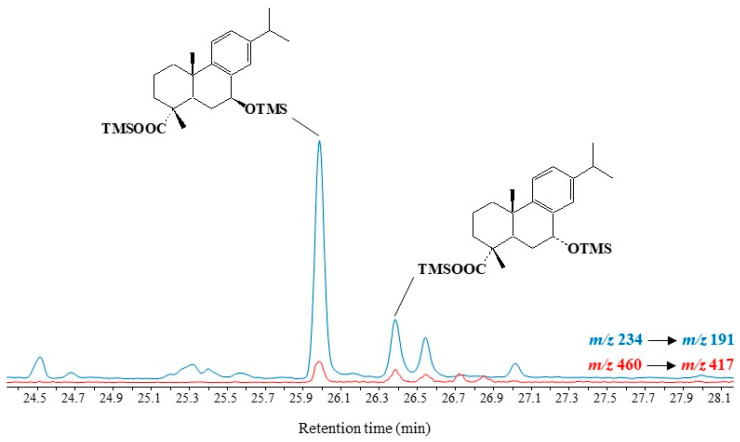
Partial MRM chromatogram (*m*/*z* 234 → 191 and *m*/*z* 252 → 237) showing the presence of autoxidation products of dehydroabietic acid in senescent needles of *Pinus halepensis*.

Fragment ions at *m*/*z* 191.0887, 234.1435 and 237.1638 can be used in GC-QTOF analyses to characterize TMS derivatives of 7α/β-hydroxydehydroabietic acids. However, MRM analyses using the highly specific transitions *m*/*z* 234 → 191 and m/z 460 → 417 [M − isopropyl group]^+^ emerged as better suited to detecting traces of these compounds in environmental samples (Figure 8).

### 3.6. Highly Branched Isoprenoid (HBI) Alkenes

**Scheme 13:**
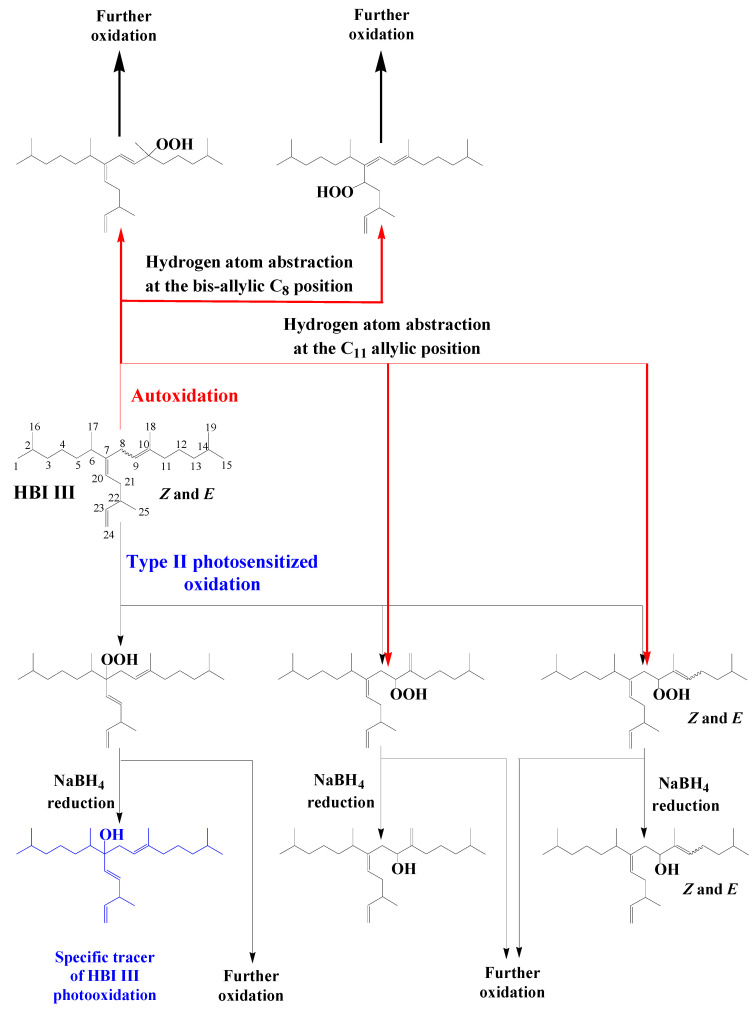
Type II photosensitized oxidation and autoxidation of HBI III.

HBI alkenes (exhibiting 1–6 double bonds) are produced by some marine and freshwater diatoms belonging to the *Berkeleya*, *Haslea*, *Navicula*, *Pleurosigma*, *Pseudosolenia* and *Rhizosolenia* genera [67,68]. During the senescence of these organisms, ^1^O_2_ attack is focused on the lesser sterically-hindered trisubstituted double bonds of these alkenes affording 2 or 4 allylic hydroperoxides according to the *E* or *Z* configuration of the double bond [69]. As an example, Scheme 13 shows type II photosensitized oxidation of *Z* and *E* isomers of HBI III, which are ubiquitous throughout the world’s oceans [70]. In this case, ^1^O_2_ attack acts mainly on the C_9_–C_10_ double bond and to a lesser extent to the more sterically-hindered C_7_–C_20_ double bond affording 9- and 7-hydroperoxides, respectively, as the major oxidation products. Autoxidation processes also act very quickly on HBI III, producing numerous autoxidation products, but predominantly 9-hydroperoxides resulting from hydrogen atom abstraction at the allylic carbon 11 (Scheme 13) [71]. Indeed, the major oxidation pathway of this compound involves hydrogen abstraction at the bis-allylic C_8_ position to afford conjugated dienes, which are particularly prone to peroxyl radical additions and readily undergo copolymerization with oxygen (Scheme 13). Consequently, the 7-alcohol resulting from NaBH_4_-reduction of the corresponding hydroperoxide could be used as specific tracer of type II photosensitized oxidation of HBI III (Scheme 13). However, the reduction products of 9-hydroperoxides will only be indicative of oxidation of this specific HBI alkene. Unfortunately, in the case of HBI alkenes (such as HBI III) possessing several trisubstituted double bonds, photooxidation and autoxidation products are unable to accumulate due to the involvement of fast secondary oxidation reactions [71]. All these tracers can thus only serve to give qualitative indications.

**Scheme 14:**
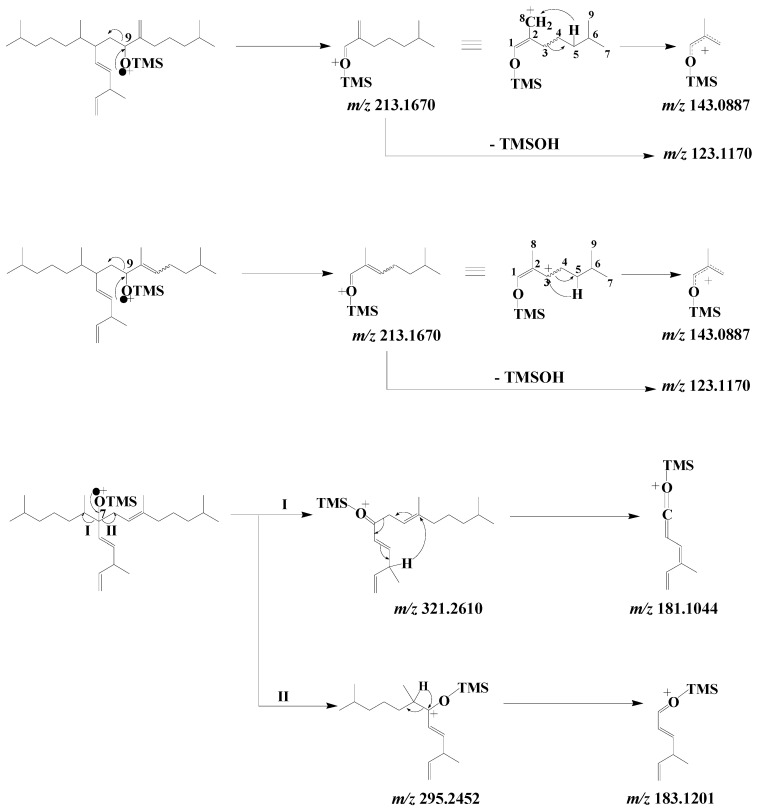
Main EI mass fragmentations of TMS derivatives of 9- and 7-alcohols resulting from oxidation of HBI III.

EI mass spectra of the TMS derivatives of the 9-alcohols resulting from HBI III oxidation exhibit an intense fragment ion at *m*/*z* 213.1670 corresponding to α-cleavage relative to the TMS ether group [72] (Scheme 14). This fragment ion can readily lose a neutral molecule of TMSOH to give a fragment ion at *m*/*z* 123.1170, or undergo a hydrogen transfer with concerted cleavage of the bond between carbon atoms 3 and 4, yielding a fragment ion at *m*/*z* 143.0887 (Scheme 14). In the case of the 7-alcohol, α-cleavage relative to the TMS ether group affords two fragment ions at *m*/*z* 295.2452 and 321.2610, which are then cleaved in the α position relative to the ionized TMS ether group after hydrogen transfers to give fragment ions at *m*/*z* 183.1201 and 181.1044, respectively (Scheme 14).

**Figure 9:**
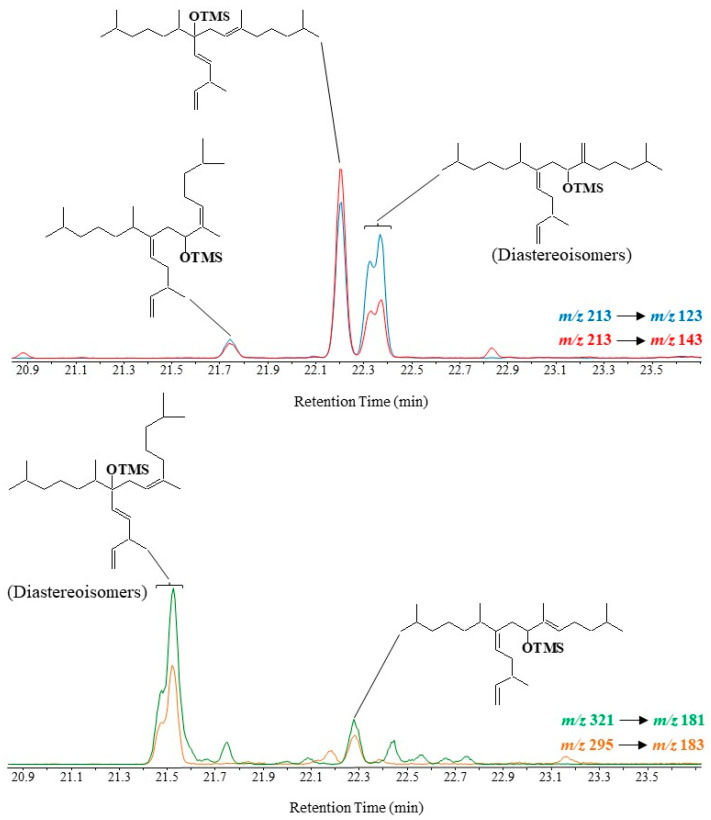
Partial MRM chromatograms (*m*/*z* 213 → 123, *m*/*z* 213 → 143, *m*/*z* 321 → 181 and *m*/*z* 295 → 183) showing the presence of oxidation products of HBI III in diatoms collected in Commonwealth Bay (Antarctic).

Oxidation products of HBI III were only characterizable in environmental samples in MRM mode using the *m*/*z* 213 → 123, *m*/*z* 213 → 143, *m*/*z* 295 → 183 and *m*/*z* 321 → 181 transitions [71,72,73]. An applied example is given in Figure 9.

### 3.7. Alkenones

Alkenones are a class of mono-, di-, tri-, tetra- and penta-unsaturated C_35_–C_40_ methyl and ethyl ketones, which are produced by certain haptophytes [74,75,76,77,78]. The unsaturation ratio of C_37_ alkenones, which is defined by the equation: $U_{37}^{K^{\prime }}$ = [C_37:2_]/([C_37:2_] + [C_37:3_]) (where [C_37:2_] and [C_37:3_] are the concentrations of di- and tri-unsaturated C_37_ methyl alkenones, respectively) varies positively with the growth temperature of the alga [79,80] and is thus now routinely used for paleotemperature reconstructions (e.g., [81,82]). Due to the *trans*- geometry of the alkenone double bonds [83], which is poorly reactive with ^1^O_2_ [33]), alkenones are not affected by type II photosensitized oxidation processes [84,85]. However, they are highly reactive to autoxidation processes [86]. Autoxidation of alkenone double bonds (separated by five or three methylene groups) affords six hydroperoxides as in the case of MUFAs (see Section 3.3). Isomeric alkenediols resulting from NaBH_4_ reduction of these oxidation products could make very useful indicators of autoxidative alterations of the unsaturation ratio $U_{37}^{K^{\prime }}$, but unfortunately they fail to accumulate due to the subsequent oxidation of the other double bonds [87]. Note that TMS derivatives of alkene-triols, tetraols or pentaols obtained after NaBH_4_ reduction and derivatization of secondary oxidation products of di-, tri- or tetraunsaturated alkenones are too heavy and labile to be analyzed by GC-MS. The characterization of alkenone autoxidation products in sediments or phytodetritus with more adapted analytical techniques constitutes a very important challenge. 

### 3.8. Carotenoids

**Scheme 15:**
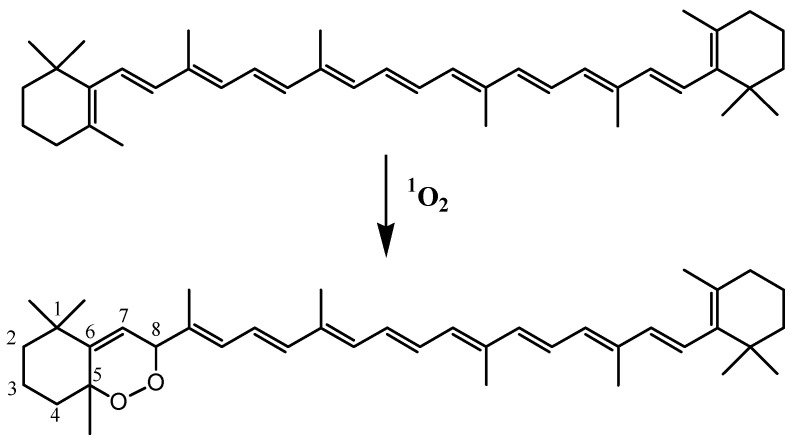
Reaction of ^1^O_2_ with β-carotene.

Carotenoids, which are important antioxidant constituents of thylakoid membranes, play special roles in the protection of tissues against damage caused by light and oxygen [88]. These compounds can very efficiently quench ^1^O_2_ by energy transfer (quenching), but also by chemical reaction (scavenging) [89]. They are also good scavengers of ROS [90]. The attack of β-carotene by ^1^O_2_ affords β-carotene-5,8-endoperoxide (Scheme 15) [91]. If this compound is generally considered a useful early signal of ^1^O_2_ production in plant leaves [92], it may be also formed during autoxidation of β-carotene [89] and is clearly not stable enough to serve as a viable environmental tracer. Unfortunately, the reaction of ^1^O_2_ and ROS with carotenoids produces oxidation products that are not sufficiently stable and specific (production of similar compounds by enzymatic processes) [89] to be used as unequivocal indicators of type-II photosensitized oxidation or autoxidation of carotenoids in senescent phototrophic organisms and environmental samples.

## 4. Conclusions

In this review, a focus was given to the selection and characterization of stable and specific tracers of photooxidation and autoxidation of lipid components (chlorophyll phytyl side-chain, ∆^5^-sterols, MUFAs, pentacyclic triterpenes and dehydroabietic acid) of phototrophs. The author hope that it will contribute to a better consideration of photooxidative and autoxidative processes almost ignored so far in the literature when studying the degradation of autotrophic organisms in marine and terrestrial environments.

The different oxidation products selected could be used as indicators of: (i) oxidative stress of specific phototrophic organisms; (ii) paleoenvironmental changes of the conditions of sedimentation (oxic or anoxic); (iii) abiotic alteration of paleoproxies in oxic environments; (iv) environmental problems related to ozone depletion, and (v) abiotic degradation of permafrost released under the effect of global warming [60].

In the future, a special attention should be given to the detection of oxidation products of alkenones and HBI alkenes (possessing several trisubstituted double bonds) sufficiently stable and specific to act as tracers of oxidative alteration of these proxies in oxic environments (water column of oceans and oxic layer of sediments). MALDI-MS and IM-MS techniques, which allow simultaneous characterization of all molecular species in biological tissues and reduce sample preparation artifacts arising from extensive purification procedures [93], seem to be particularly well-adapted to this task.

NICI GC-MS, HPLC-MS and IM-MS techniques should be also used to give evidence of the presence of isoprostanoids resulting from PUFA oxidation in environmental samples. Due to the very high reactivity of PUFA towards photooxidation and autoxidation processes, such compounds could be very sensitive tracers of the early stages of oxidative damages.

## Figures and Tables

**Table 1:** Accurate masses of the [M − 143.0887]^+^ fragment ion of TMS derivatives of ∆^4^-stera-3β,6β-diols arising from the more common ∆^5^-sterols.

∆^5^-Sterols	[M − 143.0887]^+^
24-Nor-cholesta-5,22-dien-3β,6α/β-diols	387.3084
24-Nor-cholest-5-en-3β,6α/β-diols	389.3240
Cholesta-5,22-dien-3β,6α/β-diols	401.3240
Cholesta-5,24-dien-3β,6α/β-diols	401.3240
Cholest-5-en-3β,6α/β-diols	403.3396
24-Methylcholest-5-en-3β,6α/β-diols	417.3562
24-Methylcholesta-5,22-dien-3β,6α/β-diols	415.3396
24-Methylcholesta-5,24/28-dien-3β,6α/β-diols	415.3396
24-Ethylcholest-5-en-3β,6α/β-diols	431.3710
24-Ethylcholesta-5,22-dien-3β,6α/β-diols	429.3552

**Table 2:** Accurate masses of the main fragment ions produced during EI fragmentation of silylated allylic hydroxy acids resulting from NaBH_4_-reduction of photo- and autoxidation products of some common MUFAs.

MUFAs	(OH-Position) *m*/*z*	(OH-Position) *m*/*z*	(OH-Position) *m*/*z*	(OH-Position) *m*/*z*
C_16:1∆9_	(9-) 199.1518 ^a^	(8-) 213.1675 ^a^	(10-) 329.1968 ^b^	(11-) 343.2125 ^b^
C_16:1∆11_	(11-) 171.1206	(10-) 185.1363	(12-) 357.2280	(13-) 371.2437
C_18:1∆9_	(9-) 227.1830	(8-) 241.1987	(10-) 329.1968	(11-) 343.2125
C_18:1∆11_	(11-) 199.1518	(10-) 213.1675	(12-) 357.2280	(13-) 371.2437
C_20:1∆9_	(9-) 255.2139	(8-) 269.2295	(10-) 329.1968	(11-) 343.2125
C_20:1∆11_	(11-) 227.1830	(10-) 241.1987	(12-) 357.2280	(13-) 371.2437
C_22:1∆9_	(9-) 283.2451	(8-) 297.2607	(10-) 329.1968	(11-) 343.2125
C_22:1∆11_	(11-) 255.2139	(10-) 269.2295	(12-) 357.2280	(13-) 371.2437

^a^ Fragments containing the terminal methyl group. ^b^ Fragments containing the trimethylsilyl ester group.

## Data Availability

Not applicable.

## Sample Availability

Samples of the different oxidation products (produced in very low amounts) are not available from the author, but can be easily synthesized according to the references cited.
